# Psychosocial interventions for carers of people with severe mental and substance use disorders: a systematic review and meta-analysis

**DOI:** 10.1192/j.eurpsy.2023.2472

**Published:** 2023-11-24

**Authors:** Gaia Sampogna, Elaine Brohan, Mario Luciano, Neerja Chowdhary, Andrea Fiorillo

**Affiliations:** 1 Department of Mental Health, University of Campania “L. Vanvitelli”, Naples, Italy; 2 WHO Collaborating Centre for Research and Training, Naples, Italy; 3 Department of Mental Health and Substance Use, World Health Organization, Geneva, Switzerland

**Keywords:** burden, carers, psychosocial interventions, quality of life, severe mental disorders

## Abstract

**Background:**

Severe mental disorders – such as schizophrenia, bipolar disorder, and substance use disorders – exert a negative impact not only on affected people but also on their carers. To support carers of people with severe mental disorders, several psychosocial interventions have been developed.

**Methods:**

This systematic review and meta-analysis aimed to assess whether psychosocial interventions for carers of persons with schizophrenia, bipolar disorder, or substance use disorders produce benefit/harm with respect to a series of outcomes – including subjective and objective burden, depressive symptoms, well-being/quality of life, sleep, skills/knowledge, self-efficacy, physical health – as compared to standard support/support as usual or other control conditions.

**Results:**

In carers of persons with schizophrenia, psychoeducational interventions were associated with significant improvement in personal burden, well-being, and knowledge about the illness; and a supportive-educational intervention with an improvement in personal burden. In carers of persons with bipolar disorder, psychoeducational interventions were associated with significant improvement in personal burden and depressive symptoms; family-led supportive interventions with an improvement in family burden; family-focused intervention and online “mi.spot” intervention with a significant reduction in depressive symptoms. Psychosocial interventions used for carers of persons with substance use disorders were found to be overall effective on the level of well-being, but the low number of trials did not allow detection of differences between the various psychosocial interventions.

**Conclusions:**

The quality of the evidence ranged from very low to moderate, suggesting the need for further better-quality research.

## Introduction

Severe mental disorders – such as schizophrenia, bipolar disorder, and substance use disorders – have a negative impact not only on affected people but also on their family members, carers, and the society at large. Carers often play a central role in the process of patients’ referral to mental health services, in promoting the maintenance of regular contact of patients with those services, and in supporting patients’ adherence to prescribed treatments [1–3]. Carers of people with bipolar disorder spend at least 3.9 h per day in their care, which increases to at least 5.7 h per day in carers of people with schizophrenia. Although the needs of carers of patients suffering from schizophrenia, bipolar disorder, or substance abuse disorders can be very diverse, they also face similar burdens in the taking care process. In fact, differences can be due to the specific type of illness, but also – in the case of addiction – to the abused substance (e.g., cannabinoids, opiates, and cocaine). It is also different if a person is suffering from comorbid severe mental illness and co-morbid substance use disorders because comorbidity poses an additional burden on both patients and family members [4].

Carers often report high levels of objective (i.e., the time and finances devoted to care) and subjective burden (i.e., reduced quality of life, feelings of guilt, anger, anxiety, and stress). Many carers are often unable to work or have to take time off work to provide care. Up to two billion carers work up to 8 h per day with no remuneration, with unpaid care being equal to 5% of global gross domestic product. The indirect costs of caregiving account for $112.3 billion per year, representing the main source of costs for people with severe mental disorders [5–9].

To prevent or reduce the negative impact of burden on carers of people with severe mental disorders, several psychosocial interventions are available, including psychoeducation, cognitive-behavioral therapy, counseling, and self-help groups [10–12]. There is a recognized need to support carers of people suffering from other non-communicable conditions, such as dementia [13], and to address their needs by individualized interventions [14, 15]. The global dementia action plan on the public health response to dementia 2017–2025 aims to achieve by 2025 the target of 75% of countries providing support for carers and families of people with dementia [16–20]. The implementation of the Action Plan includes delivering multisectorial interventions, including personalized psychosocial interventions for promoting the mental health and well-being of carers of people with dementia [21].

Based on this premise, the WHO has highlighted the need to provide guidance on effective psychosocial interventions to carers of people with schizophrenia, bipolar disorder, and substance use disorders, as part of the mhGAPMental Health Gap Action Programme (mhGAP). The present systematic review and meta-analysis is intended to support the development of guidance on evidence-based psychosocial interventions for carers of people with severe mental disorders. The primary aim of our systematic review and meta-analysis is to assess the impact of psychosocial interventions for carers of persons with schizophrenia/psychosis/schizophrenia spectrum disorder, bipolar disorder, or substance use disorders on the levels of carers’ subjective and objective burden, compared to standard care/usual care or other control conditions. Secondary outcomes include the effect of the psychosocial interventions on the levels of depressive symptoms, well-being/quality of life, sleep, skills/knowledge, self-efficacy, and physical health in carers of persons with schizophrenia/psychosis/schizophrenia spectrum disorder, bipolar disorder or substance use disorder.

## Methods

The following keywords were entered in the databases of PubMed/Medline, EMBASE, PsychINFO, Cochrane Central Register of Controlled Trials (CENTRAL), CINAHIL, Scopus, African Index Medicus, Index Medicus for the Eastern Mediterranean Region, Index Medicus for the South-East Asian Region, Latin American and Caribbean Health Sciences Literature, and Western Pacific Region Index Medicus: “psychosocial intervention(s),” “psychoeducation,” “cognitive-behavioral intervention(s),” “psychoeducational intervention(s),” “counselling,” “self-help,” “family member(s),” “carer(s),” “caregiver(s),” “sibling(s),” “parent(s),” “relative(s),” “spouse,” “mental disorder(s),” “schizophrenia,” “psychosis,” “alcohol use disorder(s),” “drug use disorder(s),” “severe mental illness,” “bipolar disorder,” and “family interventions.” Furthermore, repositories of systematic review protocols – including PROSPERO, Open Science Framework (OSF), and Cochrane – were searched using the same keywords. Only articles written in English were included.

The search strategy was limited to the period from 2015 to 2023 since earlier studies had been already covered in a previous systematic review [22]. Studies identified in the previous review have also been considered in the present systematic review. The AMSTAR tool was used to assess the quality of that systematic review, and the evaluation report is available in the Supplementary Material of the present paper [23].

Two researchers independently extracted the information regarding design, sample characteristics, and type of intervention for each selected study. The quality and level of evidence of each study were independently assessed by two researchers using the Grading of Recommendations Assessment, Development and Evaluation (GRADE) approach for quantitative studies [24], and the Critical Appraisal Skills Program (CASP) tool for qualitative research [25]. The GRADE approach uses a structured method for assessing the overall study quality for each outcome by one of four ratings (high, moderate, low, and very low level of certainty) based on an evaluation of risk of bias, inconsistency, indirectness, and imprecision.

Meta-analysis was performed using Review Manager (RevMan) version 5.2 for Windows. For continuous outcomes, standardized mean differences (SMDs) and 95% confidence intervals (CIs) were calculated. When studies reported data in multiple formats, SMD and its standard error were calculated before entering data in RevMan. Statistical heterogeneity was assessed by assessing *X* ^2^ value and by calculating the *I* ^2^ statistic, which describes the percentage of observed heterogeneity that would not be expected by chance. If *X* ^2^ was less than 0.05 and *I* ^2^ ≥ 60%, we considered heterogeneity to be substantial. Researchers independently assessed the studies against these criteria and resolved any discrepancy through discussion.

The following criteria have been considered: (1) carers were defined as relatives or friends who provide informal and regular care/support to someone with schizophrenia, bipolar disorder, or substance use disorders; (2) interventions were considered if they were provided to the carer of patients suffering from schizophrenia, bipolar disorder or substance use disorders; (3) primary outcomes included subjective or objective burden; and (4) secondary outcomes included quality of life, depressive symptoms and/or well-being, sleep, skills/knowledge, self-efficacy, chronic stress (e.g., measured by cortisol levels), and physical health.

Papers were excluded when benefits of psychosocial interventions were reported for the person suffering from severe mental illness, without any data on the carer. Studies focusing on carers of persons suffering from other mental disorders were excluded.

The following study designs were included: case/control study, pre/post studies, randomized controlled trials (RCTs). Non-original research, such as systematic reviews, meta-analyses, and narrative reviews, was excluded.

## Results

### Overview of the included studies

**Figure 1.**
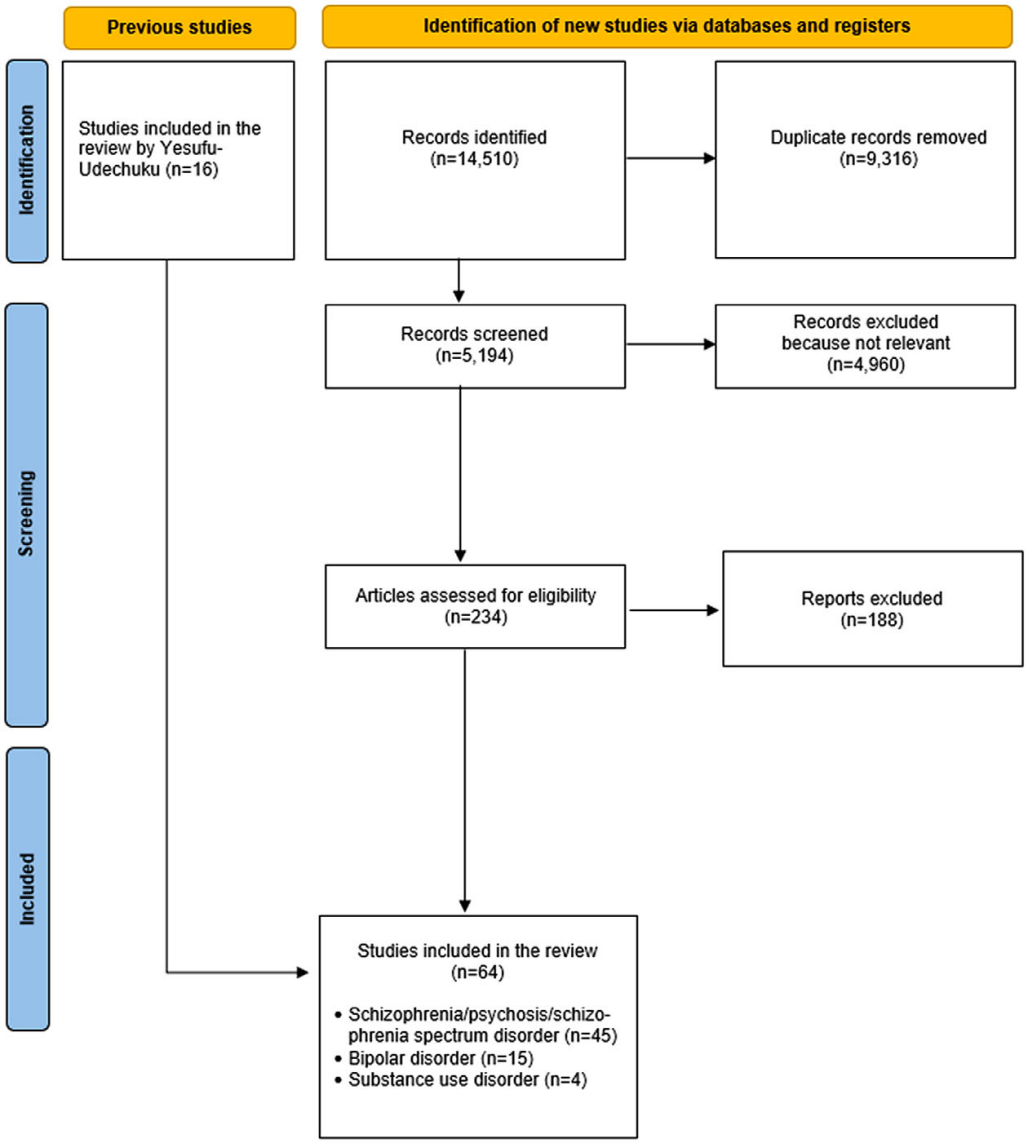
PRISMA flowchart.

The selection process of the included studies has been reported in Figure 1. Overall, 14,510 studies were retrieved from the electronic search. Of these, 9,316 were duplicates and were subsequently excluded. Of the remaining 5,194 studies, 234 full-text articles were analyzed for potential inclusion in the review. Sixteen additional papers were added based on the previous review by Yesufu-Udechuku et al. [22]. One hundred and eighty-eight papers were excluded due to non-relevant samples or outcomes, or because they were duplicated. Sixty-four studies were finally included.

Interventions for carers of persons with schizophrenia/psychosis/schizophrenia spectrum disorder were conducted in 18 countries, most frequently in Hong Kong (*n =* 8), Australia (*n =* 7), India (*n =* 5), and the UK (*n =* 4). Interventions for carers of persons with bipolar disorder were conducted in 12 countries, mainly Italy (*n =* 3), Spain (*n =* 2), Australia (*n =* 2) and the US (*n =* 2). Two out of four studies on carers of persons with substance use disorders were carried out in Iran, and the other two in the US and Australia, respectively.

The most frequently adopted study design was RCT (*n =* 48 studies; 72.7%), particularly for studies dealing with schizophrenia/psychosis/schizophrenia spectrum disorder (*n =* 34; 72.3%) and bipolar disorder (*n =* 12; 80%). Two studies (50%) on carers of persons with substance use disorders were RCTs.

Psychoeducation – defined as a psychosocial intervention with systematic and structured knowledge transfer about an illness and its treatment, integrating emotional and motivational aspects to enable carers to cope with the illness and to improve patients’ treatment adherence – was included in 38 studies on carers of persons with schizophrenia/psychosis/schizophrenia spectrum disorder, and in 14 studies on carers of persons with bipolar disorder. Psychoeducation was not used in any of the identified studies on carers of persons with a substance use disorder.

Almost all studies did not adopt rigid inclusion criteria regarding the relationship between carers and patients. The study by Koolaee and Etemdai [26] included only the mothers of patients with schizophrenia. The studies on carers of persons with substance use disorders were limited to patients’ wives [27], spouses [28, 29] or children [30].

The outcome measures most frequently considered in the various studies involved family burden (e.g., the Zarit Caregiver Burden Scale, ZCBS; the Family Assessment Device, FAD); carers’ coping strategies (e.g., the Family Coping Questionnaire, FCQ; the Coping Orientation to Problems Experienced, COPE); quality of life (e.g., the WHO-QOL-BREF; the WHOQOL-100); well-being (e.g., the Carer Wellbeing and Support, CWS; the Experience of Caregiving Inventory, ECI); and levels of knowledge (e.g., the Knowledge About Schizophrenia Interview, KASI; the Illness Perception Questionnaire for Schizophrenia-Relatives, IPQS-R; the Mental Health Literacy Scale, MHLS; the Brief Illness Perception Questionnaire, Brief IPQ).

In the majority of the included studies (*n =* 56, 84.8%), a significant positive effect of interventions on outcome measures was found. Three studies on carers of persons with schizophrenia/psychosis/schizophrenia spectrum disorder [31–33], and one study on carers of persons with bipolar disorder [34], found a modest positive effect. A positive effect of the interventions was not found in three studies on carers of persons with schizophrenia/psychosis/schizophrenia spectrum disorder [35–37], and in three studies on carers of persons with bipolar disorder [38–40].

### Studies on carers of persons with schizophrenia/psychosis/schizophrenia spectrum disorder

Forty-five studies on carers of persons with schizophrenia/psychosis/schizophrenia spectrum disorder were identified (Table 1).

**Table 1.** Studies on carers of persons with schizophrenia/psychosis/schizophrenia spectrum disorder (*n =* 45)

Author(s), country (year)	Sample and design	Intervention/control	Main components of intervention(s)	Assessment instruments	Main results in the experimental group	Global comment
Amaresha et al., India (2018) [66]	*N =* 80 siblings of persons with schizophrenia (*N =* 40 experimental intervention; *N =* 40 control group) Prospective controlled open-label trial	Brief psychoeducation program versus treatment-as-usual	Information about the disorder Drug compliance Expressed emotion Healthy lifestyles Coping strategies Practical support	Knowledge About Schizophrenia Interview (KASI) Affiliate Stigma Scale (Self-Stigma Scale) Burden Assessment Schedule (BAS)	Significant increase in knowledge and reduction in self-stigma with medium effect size.	Positive effect
Ata and Doğan, Turkey (2017) [60]	*N =* 61 carers (including mothers, fathers, siblings, spouses, others; no specific inclusion criteria) of persons with schizophrenia (*N =* 36 experimental intervention; *N =* 35 control group) Pre-test/post-test	Brief Cognitive Behavioral Stress Management Program (BCBSMP) versus treatment-as-usual	Stress management techniques Cognitive-behavioral therapy techniques Information about the disorder	General Health Questionnaire-28 (GHQ-28) Stress Self-Assessment Checklist (SIS) Scale of evaluation of coping attitude (COPE) Zarit Caregiver Burden Scale (ZCBS)	Increase in the skills related to problem-focused and emotion-focused coping. Stress indicators and levels of care burden decreased at the end of the intervention.	Positive effect
Brown and Weisman de Mamani, USA (2018) [82]	*N =* 175 carers (including mothers, fathers, siblings, spouses, others; no specific inclusion criteria) of persons with schizophrenia (*N =* 98 CIT-S group; *N =* 77 PSY-ED group) Randomized controlled trial	Culturally Informed Family Therapy for Schizophrenia (CIT-S) versus Family Psychoeducation (PSY-ED)	CIT-S Communication skills Problem-solving Coping strategies PSY-ED Standard sessions of family psychoeducation	Depression Anxiety and Stress Scale (DASS)	Reduction in individual DASS, from baseline to termination. CIT-S increased family cohesion from baseline to midpoint.	Positive effect
Budiono et al., Indonesia (2021) [93]	*N =* 64 carers (including mothers, fathers, siblings, spouses, others; no specific inclusion criteria) of persons with schizophrenia (*N =* 32 experimental intervention; *N =* 32 control group) Randomized controlled trial	Educational video materials about schizophrenia versus waiting list	Information about the disorder Current therapies Expressed emotion	Illness Perception Questionnaire for Schizophrenia – Relatives (IPQS-R) Five-Minute Speech Samples (FMSS) for evaluating family members’ expressed emotion	Positive impact on illness perception and levels of expressed emotion.	Positive effect
Bulut et al., Turkey (2016) [58]	*N =* 62 carers (including mothers, fathers, siblings, spouses, others; no specific inclusion criteria) of persons with schizophrenia (*N =* 30 experimental intervention; *N =* 32 treatment-as-usual group) Pre-test/post-test	Brief group psychoeducation versus treatment-as-usual	Information about the disorder Communication skills Problem-solving techniques Coping strategies	Perceived Family Burden Scale (PFBS)	Significant reduction in perceived family burden.	Positive effect
Chien et al., Hong Kong (2016) [44]	*N =* 116 carers (including mothers, fathers, siblings, spouses, others; no specific inclusion criteria) of persons with recent onset psychosis (*N =* 58 experimental intervention; *N =* 58 treatment-as-usual group) Randomized controlled trial	Self-help problem-solving-based manual-guided self-learning program (in addition to usual care) versus usual family support service only	Problem-solving techniques Information about the disorder	Family Burden Interview Schedule (FBIS) Experience of Caregiving Inventory (ECI) Social Problem-Solving Inventory, Revised: Short version (SPSI-R:S)	Significant improvement in ECI score and family burden.	Positive effect
Chien et al., Hong Kong (2018) [45]	*N =* 201 carers (including mothers, fathers, siblings, spouses, others; no specific inclusion criteria) of persons with recent onset psychosis (*N =* 70 Family-Led Mutual Support Group; *N =* 70 psychoeducation; *N =* 70 control group) Randomized controlled trial	Family-Led Mutual Support Group (FMSG) program (in addition to routine psychiatric outpatient care) versus psychoeducation versus treatment-as-usual	FMSG Information about the disorder Problem-solving techniques Caregiving skill practices Psychoeducation Information about the disorder Psychological support	Family Burden Interview Schedule (FBIS) Family Support Services Index (FSSI) Family Assessment Device (FAD)	Improvement of family functioning and reduction of perceived burden over a long-term follow-up with both interventions.	Positive effect
Chien et al., Hong Kong (2020) [46]	*N =* 114 carers (including mothers, fathers, siblings, spouses, others; no specific inclusion criteria) of persons with recent onset psychosis (*N =* 38 Problem-Solving Based Self-Learning Program; *N =* 38 Family Psychoeducation Group Program; *N =* 38 usual psychiatric care) Randomized controlled trial	Problem-Solving Based Self-Learning Program (PBSP), in addition to usual care (5 months) versus Family Psychoeducation Group Program (FPGP) versus usual psychiatric care and family support	PBSP Self-directed cognitive and behavioral process FPGP Information about the disorder Caregiving skills training Psychological support	Family Burden Interview Schedule (FBIS) Experience of Caregiving Inventory (ECI) Social Problem-Solving Inventory, Revised: Short version (SPSI-R:S)	Improvement in family burden and problem-solving ability.	Positive effect
Chien et al., China (2008)	*N =* 76 carers of persons with schizophrenia (*N* = 38 mutual support; *N =* 38 control) Randomized controlled trial with a repeated measures design	Peer-Led Mutual Support Group Intervention versus routine family care	Educational component Teaching coping strategies and care-giving skills	Family Burden Interview Schedule (FBIS) Six-Item Social Support Questionnaire (SSQ6)	The family burden score of the mutual support group decreased significantly over 12 months. The satisfaction of social support score of the support group increased significantly	Positive effect
Chien et al., Hong Kong (2004) [41]	*N =* 48 relatives of persons with schizophrenia (*N =* 24 experimental group; *N =* 24 control group) Randomized controlled trial	Mutual support group and usual outpatient care versus usual outpatient care	Encouraging mutual support Teaching problem-solving skills	Family burden interview schedule (FBIS) Family assessment device (FAD) Family support service index (FSSI)	Family caregivers in the mutual support group experienced a significant reduction in family burden in relation to caring for their relative with schizophrenia	Positive effect
Chien et al., China (2008)	*N =* 76 carers of persons with schizophrenia (*N =* 38 mutual support group; *N =* 38 standard care) Randomized controlled trial with a repeated measures design	Family-Led Mutual Support Group versus standard psychiatric care	Information about mental illness, treatment and community resources Emotional support and empathy	Family Burden Interview Schedule (FBIS) Family Assessment Device (FAD) Six-Item Social Support Questionnaire (SSQ6) Family Support Service Index (FSSI)	The mutual support group experienced significantly greater improvements in families’ burden, functioning and number of support persons	Positive effect
Chou et al., Taiwan (2002) [62]	*N =* 70 relatives of persons with schizophrenia (*N =* 35 experimental group; *N =* 35 control group) Non-equivalent control group design	Professionally lead support group versus no intervention	Information about community resources, financial issues pertaining to mental illness, in-home services and medical needs	Caregiver burden Inventory (CBI) Beck Depression Inventory (BDI) *Ad-hoc* participants’ satisfaction questionnaire Physical Self-Maintenance Scale (PSMS) Instrumental Activities of Daily Living (IADL) Caregiving Self-Efficacy Scale (CSS)	The depression was statistically reduced from the pre-test to the post-test and 1-month follow-up. Caregivers’ level of perceived burden changed substantially over a 8-week period of support groups and one-month follow-up.	Positive effect
Day et al., Australia (2017) [35]	*N =* 17 carers (including mothers, fathers, siblings, spouses, others; no specific inclusion criteria) of persons with early psychosis Pre-test/post-test	Journey to Recovery group program	Psychoeducation Support	*Ad-hoc* questionnaire evaluating levels of knowledge in: understanding of psychosis, recovery, medication, relapse prevention, links between substance use and psychosis	Significant improvements in family members’ understanding of psychosis, recovery, medication, relapse prevention and substance use comorbidities. Reduction in the levels of isolation and experience of stigma.	Limited effect
de Mamani and Suro, USA (2016) [65]	*N =* 113 carers (including mothers, fathers, siblings, spouses, others; no specific inclusion criteria) of persons with schizophrenia (*N =* 64 randomized to CIT-S; *N =* 49 randomized to PSY-ED) Randomized controlled trial	Family Focused, Culturally Informed Treatment for Schizophrenia (CIT-S) versus three-session psychoeducation	CIT-S Cognitive behavioral techniques Modules on spirituality and family collectivism Information on the disorder (causes, treatments, consequences)	Shame and guilt/self-blame assessed using Likert ratings that ranged from 1 to 7 Modified Burden Assessment Scale for Families of the Seriously Mentally Ill (BAS)	CIT-S was found to outperform PSY-ED in reducing guilt/self-blame and caregiver burden.	Positive effect
Deane et al., Australia (2015) [50]	*N =* 81 carers (including mothers, fathers, siblings, spouses, others; no specific inclusion criteria) of persons with psychosis (*N =* 40 experimental intervention; *N =* 41 control group) Randomized controlled trial	Connection group (information booklet, followed by 12 recovery-focused interactive newsletters) versus information only	Goal-directed informative booklet, focusing on strengths and promoting personal growth and development	Kessler-10 (K10) Experience of Caregiving Inventory (ECI) Psychological Well-Being (PWB) Adult State Hope Scale (ASHS) Recovery Knowledge Inventory (RKI)	Improvements in distress, hope and negative caregiving experiences over 12 months.	Positive effect
Friedman-Yakoobian et al., USA (2016) [59]	*N =* 10 carers (including mothers, fathers, siblings, spouses, others; no specific inclusion criteria) of persons with schizophrenia Pre-test/post-test	Family Directed Cognitive Adaptation (including psychoeducation about schizophrenia and related cognitive difficulties; feedback about the client’s cognitive strengths and weaknesses; and collaborative identification of cognitive adaptation strategies)	Psychoeducation Problem-solving techniques	Burden Assessment Scale for Families of the Seriously Mentally Ill (BAS) Client Satisfaction Questionnaire (CSQ)	Significant reduction in burden on the BAS at the end of treatment, which was maintained at follow-up.	Positive effect
Gleeson et al., Australia (2017) [51]	*N =* 29 carers (including mothers, fathers, siblings, spouses, others; no specific inclusion criteria) of persons with first episode psychosis Pre-test/post-test	Moderated Online Social Therapy (MOST)	Self-care Psychoeducation Communication skills Coping strategies	Perceived Stress Scale (PSS) Depression Anxiety and Stress Scale (DASS) Scales of Psychological Wellbeing Medical Outcomes Study: Social Support Survey (MOS-SSS)	Moderate correlations between reductions in stress and use of the online system. Moderate and significant correlations between degree of improvement in stress and number of log-ons.	Positive effect
Gutiérrez-Maldonado and Caqueo-Urìzar, Chile (2007) [68]	*N =* 45 caregivers of persons with schizophrenia (*N =* 22 psycho-educational family intervention group; *N =* 23 control group) Randomized controlled trial	Psycho-educational family program versus standard intervention	Family’s experience of schizophrenia Psycho-education Skills to improve communication Relatives’ self-care	Zarit Caregiver Burden Scale (ZCBS) *Ad-hoc* caregivers’ knowledge of schizophrenia questionnaire	Burden decreased significantly in the psychoeducational group	Positive effect
Hamza et al., India (2019) [36]	*N =* 48 carers (including parents, siblings, spouses, children) of persons with schizophrenia (*N =* 23 experimental intervention; *N =* 25 control group) Randomized controlled trial	Self-help yoga manual associated with visual support (DVD) versus usual care	Video and photo shoot of yoga procedures	Burden Assessment Scale (BAS) Perceived Stress Scale (PSS) WHO Quality Of Life Scale (WHOQOL-Brief)	No changes in burden, stress and quality of life at the end of the intervention.	No effect
Koolaee and Etemdai, Iran (2009) [26]	*N =* 55 mothers of persons with schizophrenia (*N =* 18 behavioral family group; *N =* 19 psychoeducation group; *N =* 18 standard care Randomized controlled trial	Psychoeducation group versus behavioral family management group versus group receiving standard psychiatric care	Psychoeducation Information on the disorder Problem-solving training Behavioral family management group intervention Information on the disorder Communication skills Standard outpatient care Counselling	Family Burden Interview Schedule (FBIS) Family Questionnaire (FQ)	The perceived burden reduced significantly over time when compared with the score for the behavioral family management group	Positive effect
Kordas et al., Poland (2015) [57]	*N =* 13 carers (including parents and siblings) of persons with schizophrenia Pre-test/post-test	Psychoeducation	Information about the disorder Communication skills Psychodrawing	*Ad-hoc* questionnaire including: participants’ needs and expectations; knowledge of schizophrenia and its treatment; stress and illness-related burden	No significant increase in participants’ theoretical knowledge on schizophrenia. Reduced subjective sense of burden in the family.	Positive effect
Kumar et al., India (2020) [67]	*N =* 66 key carers (various) of persons with schizophrenia (*N =* 33 experimental intervention; *N =* 33 nonspecific control intervention group) Randomized controlled trial	Brief psychosocial intervention, consisting of two sessions of psychoeducation on individual basis, followed by six group therapy sessions, versus general information and support only	Information about the disorder Expressed emotion Problem-solving techniques Coping strategies Communication skills	Burden Assessment Schedule (BAS) WHO Quality Of Life Scale (WHOQOL-100)	Significant reduction in the levels of burden of care and improvement in quality of life.	Positive effect
Leavey et al., 2004 (UK) [54]	*N =* 106 carers of persons with FEP (*N =* 57 experimental group; *N =* 49 control group) Randomized controlled trial	Experimental versus treatment as usual (usual support from the psychiatric service)	Psychoeducation	Verona Service Satisfaction Questionnaire (Relatives) (VSSS-32) Perceived severity of illness Caregiver Strain Index (CSI)	Significant reduction in the levels of strain experienced by carers	Positive effect
Lobban et al., UK (2020) [56]	*N =* 800 carers (various, no specific inclusion criteria) of persons with either psychosis or bipolar disorder (*N =* 399 experimental intervention; *N =* 401 control group) Randomized controlled trial	REACT (psychoeducation modules, peer support through a group forum, confidential messaging and a comprehensive resource directory of national support) versus access to the same resource directory. All trial participants received treatment as usual	Psychoeducation modules Information about the disorder Stress management	Carer Wellbeing and Support (CWS) Questionnaire General Health Questionnaire-28 (GHQ-28) Brief Illness Perception Questionnaire (Brief IPQ)	Significant reduction of distress. Carer wellbeing and support both increased significantly over time. *(Not possible to separate outcomes according to patient’s diagnosis).*	Positive effect
Martìn-Carrasco et al., Spain (2016) [101]	*N =* 223 carers (including mothers, fathers, siblings, spouses, others; no specific inclusion criteria) of persons with schizophrenia (*N =* 109 experimental intervention; *N =* 114, control group) Randomized controlled trial	Psychoeducational Intervention Program (PIP) versus standard care	Behavioral-cognitive approach Information about the disorder Cognitive strategies for reframing negative emotional responses Healthy lifestyle Stress management	Zarit Burden Interview (ZBI) Involvement Evaluation Questionnaire (IEQ)	Significant reduction of family burden at 4-month and 8-month follow-up.	Positive effect
McCann et al., Australia (2017) [52]	*N =* 124 carers (mostly parents) of persons with first episode psychosis (*N =* 61 experimental intervention; *N =* 63 treatment-as-usual) Randomized controlled trial	Self-help problem-solving bibliotherapy “Reaching Out: Supporting a Family Member or Friend with First-Episode Psychosis”	Information about the disorder Problem-solving techniques Communication skills	Social Problem-Solving Inventory-Revised: Short Form (SPSI-R:S)	Improvement of problem-solving abilities, maintained at both follow-up time points.	Positive effect
McCann et al., Australia (2012) [49]	*N =* 124 relatives of persons with FEP (*N =* 61, bibliotherapy; *N =* 63, treatment as usual) Randomized controlled trial	Problem-Solving Bibliotherapy Intervention (PSBI) versus treatment as usual (specialist support, coordinated by a case manager and psychiatrist)	Problem-solving based bibliotherapy Information on strengthening the carer’s well-being (physical and mental) and coping skills	Experience of Caregiving Inventory (ECI) Kessler Psychological Distress Scale (K10) Family Questionnaire (FQ) Short Form Health Survey (SF-12)	The PSBI group experienced a greater reduction in negative emotional evaluations of the need to provide additional support to young people with FEP than the TAU group by week 6, while the level of psychological distress decreased at a greater rate from baseline to 6 weeks in the PSBI compared with the TAU group.	Positive effect
Mubin et al., Indonesia (2019) [96]	*N =* 84 carers (various, no specific inclusion criteria) of persons with schizophrenia (*N =* 42 experimental intervention; *N =* 42 control group) Randomized controlled trial	Psychoeducation versus standard educational care	Information about the disorder Stress management Coping strategies	Indonesian version of Care Burden Scale (CBS)	Significant reduction of family burden.	Positive effect
Ngoc et al., Vietnam (2016) [69]	*N =* 59 carers (various, no specific inclusion criteria) of persons with schizophrenia (*N =* 30 experimental intervention; *N =* 29 control group) Randomized controlled trial	Family Schizophrenia Psychoeducation Program (FSPP) versus treatment-as-usual	Information about the disorder Problem-solving techniques Communication skills	Quality of Life Enjoyment and Satisfaction Questionnaire (QLESQ) Stigma Toward Schizophrenia Scale developed for Vietnamese patients (STSS) *Ad-hoc* questionnaire for evaluating consumer satisfaction	Significant reduction in family-reported stigma and quality of life, with effect sizes from moderate to large.	Positive effect
Öksüz et al., Turkey (2017) [63]	*N =* 60 carers (including parents, siblings and spouses; no specific inclusion criteria) of persons with first episode psychosis (*N =* 30 experimental intervention; *N =* 30 control group) “Quasi-experimental design with control group”	Psychoeducation versus treatment-as-usual	Information about the disorder Communication skills	Expressed Emotion Scale (EES) Family Assessment Device (FAD)	Decrease of expressed emotion (such as criticism/hostility and overinvolvement-protecting), improvement in family functioning.	Positive effect
Posner et al., Canada (1992) [31]	*N =* 55 family members of persons with schizophrenia (*N =* 28 experimental condition; *N =* 27 control condition) Randomized controlled trial	Psychoeducation support-group program versus waiting list	Psychoeducational approach including educational component; coping strategies, problem-solving and communication skills	Schizophrenia Knowledge Test (SKT) Consumer Satisfaction Questionnaire (CSQ) Family Satisfaction Scale (FSS) Negative Feelings for Patients Ways of coping (WOC) General Health Questionnaire (GHQ)	Carers reported a significant improvement in the levels of knowledge on the illness and reported a more positive evaluation of healthcare services. No significant change in levels of coping strategies, family satisfaction and well-being were found.	Positive effect only on levels of burden
Puspitosari et al., Indonesia (2019) [97]	*N =* 100 carers (including mothers, fathers, siblings, spouses, others; no specific inclusion criteria) of persons with schizophrenia (*N =* 50 experimental intervention; *N =* 50 control group) “Quasi-experimental study”	Psychoeducation and social skills training versus routine outpatient care	Psychoeducation Social skills training Stress management Communication skills	Quality of Life Interview (QOLI)	Improvement in quality of life.	Positive effect
Rami et al., Egypt (2018) [98]	*N =* 50 carers (first degree relatives) of persons with schizophrenia (*N =* 26 experimental intervention; *N =* 24 control group) Randomized controlled trial	Behavioral Family Psycho-Educational Program (BFPEP) versus treatment-as-usual	Psychoeducation Communication enhancement training Skills for active listening Problem-solving	Social Functioning Questionnaire (SFQ) Quality of Life Scale (QLS) Drug Attitude Inventory (DAI)	Improvement in the levels of social functioning, attitudes toward medication, and quality of life.	Positive effect
Sharif et al., Iran (2012) [100]	*N =* 70 caregivers of persons with schizophrenia (*N* = 35 experimental group; *N =* 35 control groups) Randomized controlled trial	Psychoeducational intervention versus no intervention	Psychoeducational approach	Family Burden Questionnaire (FBQ)	Positive effects in reduction of family burden immediately and one month after the intervention.	Positive effect
Sharma et al., India (2021) [99]	*N =* 40 carers (including mothers, fathers, daughters, sons, spouses, siblings; no specific inclusion criteria) of persons with schizophrenia (*N =* 20 experimental intervention; *N =* 20 control group) Randomized controlled trial	Psychoeducation versus no intervention	Information about the disorder Communication skills Expressed emotion	Ryff Psychological Wellbeing (PWB) scale	Significant improvement in emotional regulation and in levels of personal wellbeing.	Positive effect
Shiraishi et al., Japan (2019) [37]	*N =* 74 carers (including mothers, fathers, spouses, siblings; no specific inclusion criteria) of persons with recent onset or chronic psychosis (*N =* 37 experimental intervention plus treatment-as-usual; *N =* 37 treatment-as-usual only) Randomized controlled trial	Standard Model of Family Psychoeducation (SM-FPE) versus treatment-as-usual	Information about the disorder Communication skills Problem-solving	Japanese version of Zarit Burden Interview Short version (J-ZBI-8) Family Accommodation Scale (FAS) Link’s Stigma Scale (LSS)	No effects on anxiety, family burden and levels of expressed emotions.	No effect
Smeerdijk et al., The Netherlands (2015) [64]	*N =* 97 carers (parents) of persons with schizophrenia (*N =* 53 experimental intervention; *N =* 47 control group) Randomized controlled trial	Psychoeducation followed by either Family Motivational Intervention (FMI) or Routine Family Support (RFS) versus RFS only	Psychoeducation Problem-solving techniques Motivational interview	Experience of Caregiving Inventory (ECI) Family Questionnaire (FQ) General Health Questionnaire (GHQ-28)	Both groups improved in parental distress and sense of burden. Only in the FMI group, a further decrease of parental distress was observed from 3-month to 15-month follow-up.	Positive effect
So et al., Hong Kong (2006) [43]	*N =* 55 carers of people with FEP (*N =* 22 experimental group; *N =* 23 control group) Randomized controlled trial	Experimental intervention versus waiting list control condition	Knowledge about psychosis, skills in handling the patients’ illness and their own caregiving stress; stress management, communication skills, and relapse prevention	Level of Expressed Emotion (LEE) Knowledge about psychosis scale Experience of Caregiving Inventory (ECI) Chinese Ways of Coping Questionnaire (CWCQ) Life Events Questionnaire (LEQ)	Carers significantly improved levels of knowledge about psychosis	Positive effect
Szmukler et al., Australia (1996) [32]	*N =* 63 carers of persons with schizophrenia (*N =* 32 Intervention group; *N =* 31 Control group) Randomized controlled trial	Experimental intervention: counselling Control condition: information about the illness and services	Educational component Teaching of coping Strategies	General Health Questionnaire (GHQ) Assessment of physical status Positive and Negative Affects Scale Experience of Caregiving Inventory (ECI) Mastery	Carers reported a better understanding of the patient and in the perception of a more positive relationship. No significant difference was found between the groups for global physical health.	Positive effects only on levels of knowledge
Szmukler et al., Australia (2003) [33]	*N =* 61 carers of persons with psychosis (*N =* 30 experimental intervention; *N =* 31, standard care) Randomized controlled trial	Experimental intervention versus control condition	Experimental intervention: counselling including educational and problem-solving components Control intervention: single counselling session	Experience of Caregiving Inventory (ECI) Ways of Coping (WOC) Mastery	A small effect in enhancing positive aspects of the relationship with the patient and a stronger effect in helping the relative understand the patient’s illness better were found	Slight positive effect
Verma et al., India (2019) [61]	*N =* 30 carers (first degree relatives) of persons with schizophrenia (*N =* 15 experimental intervention; *N =* 15 control group) Pre-test/post-test	Family psychoeducation versus no intervention	Information about the disorder Expressed emotion	WHO Quality Of Life Scale (WHO-QOL-Brief)	Improvement in quality of life.	Limited effect
Zhou et al., Hong Kong (2020a) [47]	*N =* 89 carers (including parents, siblings and spouses; no specific inclusion criteria) of persons with schizophrenia (*N =* 46 experimental intervention; *N =* 43 control group) Randomized controlled trial	Collective Narrative Therapy Groups (CNTG) versus waiting list	Experiential learning Role play Discussion of difficulties	Brief Family Relationship Scale (BFRS) Experience of Caregiving Inventory (ECI-66) Mental Health Inventory (MHI-5): mental wellbeing	Improvement in family relationship, caregiving experiences, inner resources, hope, mental health status and caregiving burden.	Positive effect
Zhou et al., Hong Kong (2020b) [48]	*N =* 132 carers (including parents, siblings and spouses; no specific inclusion criteria) of persons with schizophrenia (*N =* 29 psychoeducation; *N =* 34 narrative therapy; *N =* 31 control group) Randomized controlled trial	FamilyLink Education Program (FLEP), a peer-led psychoeducational program, versus narrative-based intervention versus waiting list	Information about the disorder Coping strategies Communication skills Storytelling of personal experiences	Brief Family Relationship Scale (BFRS) Experience of Caregiving Inventory (ECI) Family Coping Questionnaire (FCQ) Mental Health Inventory (MHI) Pearline Mastery Scale (PMS)	Improvement of caregiving burden in both intervention groups.	Positive effect

The studies were conducted most frequently in Hong Kong [41–48], Australia [32, 33, 35, 49–52], and the UK [53–56].

The majority of studies adopted an RCT design. Eight studies [30, 35, 51, 57–61] used a pre-test/post-test design, and one study adopted a non-equivalent control group design [62].

The samples mostly consisted of carers of persons with a diagnosis of schizophrenia. Four studies [44–46, 55] included carers of persons with recent onset of psychosis; seven recruited carers of persons with first-episode psychosis [35, 43, 49, 51, 52, 54, 63]; and one included carers of persons with either recent onset or chronic psychosis [37]. The study by Deane et al. [50] included carers of persons affected by psychosis, without any further specification. The study by Lobban et al. [56] included carers of persons with either psychosis or bipolar disorder.

Ten studies [32, 33, 43, 44, 46–50, 64] used the Experience of Caregiving Inventory (ECI), and five studies [36, 59, 65–67] the Burden Assessment Schedule (BAS). Only four studies [35, 57, 68, 69] used *ad-hoc* assessment tools, specifically developed for the purposes of the study.

The majority of studies implemented a psychoeducational program/approach. Five studies [36, 44, 45, 52, 55] used a self-help intervention; two studies implemented a mutual support group approach [41, 62]; and one used bibliotherapy [49]. Almost all studies reported a positive effect on considered outcomes. Three studies [31–33] found a modest positive effect, and three [35–37] found no positive effect of the experimental intervention (Table 1).

#### Efficacy of psychosocial interventions on primary and secondary outcomes in carers of persons with schizophrenia/psychosis/schizophrenia spectrum disorder

In the overall group of carers of persons with schizophrenia receiving any psychosocial intervention, there was an improvement in the levels of personal burden (standardized mean difference, SMD: –0.61, 95% CI: –0.86 to –0.36, *p <* 0.005), in well-being/quality of life (SMD: 0.72, 95% CI: 0.39 to 1.05, *p <* 0.005) and in the levels of knowledge about the disorder (SMD: 0.60, 95% CI: 0.20 to 1.01, *p <* 0.005) and self-efficacy (SMD: 1.15, 95% CI: 6.16 to 8.46, *p <* 0.005; Table 3).

**Table 2.** GRADE table for psychosocial interventions compared to treatment as usual, usual psychiatric care, or waiting list for carers of persons with schizophrenia/psychosis/schizophrenia spectrum disorder

Certainty assessment							No. of patients		Effect		Certainty	Importance
No. of studies	Study design	Risk of bias	Inconsistency	Indirectness	Imprecision	Other considerations	Psychosocial interventions	Placebo	Relative (95% CI)	Absolute (95% CI)		
*Personal Burden (assessed with: Burden Assessment Schedule or Zarit Caregiver Burden Scale or Family Burden Interview Schedule or Burden Assessment scale or Caregiver Burden Inventory or Family Burden Questionnaire)*												
22	Randomized trials	Not serious	Serious^a^	Not serious	Not serious	None	971	820	–	SMD **0.61** **SD lower** (0.86 lower to 0.36 lower)	⨁⨁⨁◯ Moderate	CRITICAL
*Wellbeing/quality of life (assessed with: General Health Questionnaire or Psychological Wellbeing or Wellbeing Medical Outcomes Study: Social Support Survey or WHOQOL-Brief or Carer wellbeing and Support Questionnaire or Quality of Life Enjoyment and Satisfaction Questionnaire or Quality of Life Interview or Quality of Life Scale or Ryff Psychological Wellbeing Scale or Mental Health Inventory)*												
18	Randomized trials	Not serious	Serious^a^	Not serious	Not serious	None	1,021	920	–	SMD **0.72 SD higher** (0.39 higher to 1.05 higher)	⨁⨁⨁◯ Moderate	CRITICAL
*Depressive symptoms (assessed with: Depression Anxiety Stress Scale (DASS) or Beck Depression Inventory (BDI) or Kessler Psychological Distress or Positive and Negative Affects Scale)*												
6	Randomized trials	Not serious	Serious^a^	Not serious	Serious^b^ ^,^^c^	None	271	247	–	SMD **0.76 SD lower** (1.61 lower to 0.1 higher)	⨁⨁◯◯ Low	CRITICAL
*Knowledge about the disorder (assessed with: Knowledge about schizophrenia interview or illness perception questionnaire for schizophrenia-relatives or ad-hoc questionnaire or knowledge about psychosis scale or Schizophrenia Knowledge Test (SKT))*												
7	Randomized trials	Not serious	Serious^a^	Not serious	Serious^c^	None	207	162	–	SMD **0.6 SD higher** (0.2 higher to 1.01 higher)	⨁⨁◯◯ Low	IMPORTANT
*Skills/coping skills (assessed with: Family Coping Questionnaire or Social Problem-Solving Inventory Revised Short Form or Brief COPE or COPE)*												
9	Randomized trials	Not serious	Serious^a^	Not serious	Serious^b^	None	754	692	–	SMD **0.1 SD higher** (0.21 lower to 0.41 higher)	⨁⨁◯◯ Low	IMPORTANT
*Self-efficacy (assessed with: General Perceived Self-Efficacy Scale (SES) or Caregiving Self- Efficacy Scale (CSS))*												
2	Randomized trials	Serious	Serious^a^	Not serious	Serious^b^	None	67	67	–	SMD **1.15 SD higher** (6.16 lower to 8.46 higher)	⨁◯◯◯ Very low	IMPORTANT

CI, confidence interval; SMD, standardized mean difference.

a Severe, unexplained, heterogeneity (*I* ^2^ ≥ 60% or X^2^ < 0.05).

b Wide CI crossing the line of no effect.

c Less than 400 participants.

Bold characters indicates statistical significance at *p* < 0.05.

**Table 3.** Studies on carers of persons with bipolar disorder (*n =* 15)

Author(s), country (year)	Sample and design	Intervention	Main components of intervention(s)	Assessment instruments	Main results in the experimental group	Global comment
Abad et al., Iran (2021) [40]	*N =* 60 wives (*N =* 30 experimental intervention; *N =* 30 control group) Randomized controlled trial	Problem-solving skills training versus psychological support	Problem-solving techniques Brain storming	Index of Spouse Abuse	Significant changes in abuse scores (physical, non-physical and total scores).	Positive effect
Barbeito et al., Spain (2021) [81]	*N =* 148 carers (various; no specific inclusion criteria; *N =* 74 experimental intervention; *N =* 74 control group) Randomized controlled trial	Multifamily psychoeducational program versus control group discussing general topic	Psychoeducation Problem-solving techniques	Family Burden Self-Report (FB-SR) scale Strauss-Carpenter Scale (SCS)	Significant improvement in objective and subjective family burden.	Positive effect
de Souza et al., Brazil (2016) [38]	*N =* 53 carers (including mothers, partners or others; no specific inclusion criteria; *N =* 25 experimental intervention; *N =* 28 control group) Randomized controlled trial	Psychoeducational intervention versus sessions with the caregiver without any specific intervention	Information about the disorder Stress management Early warning signs	Family Burden Interview Schedule (FBIS) Rosenberg Self-Esteem Scale (RSS) Health Survey-36-Item Short Form (SF-36)	No significant improvement in levels of burden, self-esteem and quality of life.	No effect
Fiorillo et al., Italy (2015) [70]	*N =* 155 carers (including parents, spouses/significant others; no specific inclusion criteria; *N =* 85 experimental intervention; *N =* 70 control group) Randomized controlled trial	Psychoeducational family intervention versus waiting list	Information about the disorder Problem-solving techniques Communication skills	Social Network Questionnaire (SNQ) Family Problem Questionnaire (FPQ)	Significant reduction of relatives’ objective and subjective burden.	Positive effect
Gex-Fabry et al., Switzerland (2015) [75]	*N =* 26 carers (including partners, fathers, mothers, brothers, sisters; no specific inclusion criteria) Pre-test/post-test study	Group psychoeducation	Information on the disorder and its treatment Problem-solving techniques	World Health Organization Quality of Life questionnaire (WHOQOL-BREF)	Benefits in detecting the early warning signs of relapse, improvement of quality of life, feeling more involved in caregiving activities.	Positive effect
Hubbard et al., Australia (2016) [73]	*N =* 32 carers (including partners, parents, siblings, friends; no specific inclusion criteria; *N =* 14 experimental intervention; *N =* 18 waiting list) Randomized clinical trial	Brief, two-session psychoeducational intervention for caregivers versus waiting list	Psychoeducation Coping strategies Communication skills Problem-solving techniques	Depression Anxiety and Stress Scale (DASS-21) Burden Assessment Scale (BAS) *Ad-hoc* scale on knowledge of bipolar disorder *Ad-hoc* scale on bipolar disorder self-efficacy	Significant reductions in burden, improvement in self-efficacy and knowledge.	Positive effect
Lobban et al., UK (2020) [56]	*N =* 800 carers (various, no specific inclusion criteria; *N =* 399, experimental intervention; *N =* 401, control group) Randomized controlled trial	REACT (psychoeducation modules, peer support through a group forum, confidential messaging and a comprehensive resource directory of national support) versus access to the same resource directory. All trial participants received treatment as usual	Information about the disorder Stress management	Carer Wellbeing and Support (CWS) Questionnaire General Health Questionnaire-28 (GHQ-28) Brief Illness Perception Questionnaire (Brief IPQ) Brief Coping Orientation to Problems Experienced inventory (Brief COPE)	Significant reduction of distress. Carer wellbeing and support both increased significantly over time.	Positive effect
Luciano et al., Italy (2015) [71]	*N =* 155 carers (including parents, spouses/significant others; no specific inclusion criteria; *N =* 85 experimental intervention; *N =* 70 control group) Randomized controlled trial	Psychoeducational family intervention versus waiting list	Information about the disorder Problem-solving techniques Communication skills	Social Network Questionnaire (SNQ) Family Problem Questionnaire (FPQ)	Reduction in objective and subjective family burden.	Positive effect
Madigan et al., Ireland (2012) [95]	*N =* 47 carers of persons with bipolar disorder (*N =* 18 Multi Family Group Psychoeducation; *N =* 19 Solution Focused Group Psychotherapy; *N =* 10 Treatment as Usual) Randomized controlled trial	Multifamily Group Psychoeducation (MFGP) versus Solution Focused Group Psychotherapy (SFGP) versus Treatment as usual (TAU)	MFGP Psychoeducation SFGP Teaching of problem-solving strategies	Knowledge of Illness Questionnaire (KOIQ) Involvement Evaluation Questionnaire (IEQ) General Health Questionnaire (GHQ12) Quality of Life (WHOQOL Bref)	Carers in both the MFGP intervention and the SFGP arm demonstrated greater knowledge and reduction in burden.	Positive effect
O’Donnell et al., USA (2020) [39]	*N =* 145 carers (various; no specific inclusion criteria; *N =* 72 experimental intervention; *N =* 72 control group) Randomized controlled trial	Psychoeducation, communication training, and problem-solving skills training versus enhanced care (briefer psychoeducational treatment)	Information about the disorder Communication skills Problem-solving techniques	Family Adaptability and Cohesion Evaluation Scale (FACES-II) Conflict Behavior Questionnaire (CBQ)	Increase in family cohesion and adaptability and decrease in family conflict.	Positive effect
Perlick et al., USA (2018) [74]	*N =* 46 carers (including parents, spouses/significant others, children, friends; no specific inclusion criteria; *N =* 25 experimental intervention; *N =* 21 control group) Randomized controlled trial	Caregiver-only adaptation of family-focused treatment (FFT) versus sessions of standard health education	Psychoeducation and goal setting Behavioral analysis of self-care barriers Cognitive behavioral therapy Problem-solving techniques	Social Behavior Assessment Scale (SBAS) Health Risk Behavior Scale (HRB)	Improvement of depressive symptoms, overall psychological health and levels of burden.	Positive effect
Reinares et al., Spain (2004) [34]	*N =* 45 carers of persons with bipolar I or II disorder (*N =* 30 experimental group; *N =* 15 control group) Randomized controlled trial	Psychoeducational Family Intervention versus no treatment	Psychoeducational family intervention Structured information about the disorder Teaching of coping strategies	Social Behavior Assessment Schedule (for family burden) Family Environment Scale Bipolar Disorder Knowledge Questionnaire	No significant changes were found in the objective burden nor in the relationships within the family environment. Improvement in caregivers’ knowledge of bipolar disorder	Some effect only on knowledge
Reupert et al., Australia (2018) [30]	*N =* 31 children (aged 18–25 years) with a parent having mental illness and/or substance use disorder Pre-test/post-test	Online intervention (“mi.spot”) targeting cognitive reappraisal, connectedness to others, and resilience	Information about the disorder Coping strategies	Mental Health Continuum Short Form (MHC-SF) Depression Anxiety and Stress Scale (DASS-21) Coping Orientation to Problems Experienced (COPE) inventory General Help-Seeking Questionnaire (GHSQ) Social Connectedness Scale (SCS) Mental Health Literacy Scale (MHLS) General Self-Efficacy Scale (GSE)	Improvement in depressive symptoms, stress levels, well-being and autonomy.	Positive effect
Sampogna et al., Italy (2018) [72]	*N =* 139 carers (including parents, spouses/significant others; no specific inclusion criteria; *N =* 72 experimental intervention; *N =* 67 control group) Randomized controlled trial	Psychoeducational family intervention versus waiting list	Information about the disorder Problem-solving techniques Communication skills	Family Coping Questionnaire (FCQ)	Improvement in problem-oriented coping strategies, such as positive communication strategy.	Positive effect
Zyto et al., Netherlands (2020) [76]	*N =* 88 carers (including parents, spouses, others; no specific inclusion criteria) Pre-test/post-test	Psychoeducational program	Psychoeducation Information about the disorder	Level of Expressed Emotion (LEE)	Significant reduction in expressed emotion.	Positive effect

Risk of bias was rated as serious only for studies considering self-efficacy as outcome measure. For the remaining outcomes (i.e., personal burden, well-being/quality of life, depressive symptoms, knowledge about the disorder, and skills/coping skills) risk of bias was not serious. Inconsistency was rated as serious for all considered outcomes, while indirectness was not serious for any of them. Imprecision was rated as serious for studies considering depressive symptoms, knowledge about the disorder, skills/coping skills, and self-efficacy as outcome measures (see Table 2). Certainty of evidence was rated as moderate for studies considering personal burden and well-being/quality of life as outcome measures; low for studies considering depressive symptoms, knowledge about the disorder, and skills/coping skills as outcome measures; and very low for studies considering self-efficacy as outcome measure.

#### Subgroup analysis on efficacy of psychosocial interventions on carers of persons with schizophrenia/psychosis/schizophrenia spectrum disorder

A subgroup analysis based on the type of psychosocial intervention included in the different studies was performed (see Supplementary Table 1). A definition of the included approaches is provided in Appendix 1. Psychoeducational intervention was significantly associated with an improvement in the levels of personal burden (SMD: –0.70, 95% CI: –1.01 to –0.40, *p <* 0.005), well-being (SMD: 0.72, 95% CI: 0.39 to 1.54, *p <* 0.005) and knowledge about the illness (SMD: 0.60, 95% CI: 0.2 to 1.01, *p <* 0.005). Supportive-educational intervention was significantly associated with an improvement in personal burden (SMD: –0.26, 95% CI: –0.67 to –0.14, *p <* 0.005), but not with other considered outcomes. The remaining types of interventions were not associated with a statistically significant improvement in considered outcomes.

### Studies on carers of persons with bipolar disorder

Fifteen studies on carers of persons with bipolar disorder were identified (Table 3), which were carried out mainly in Italy [70–72], Australia [30, 73], and the US [39, 74].

The majority of studies used an RCT design. Three studies [30, 75, 76] adopted a pre-test/post-test design. *Ad-hoc* assessment tools were used only in the study by Hubbard et al. [73], while the other studies adopted very different assessment tools (Table 3).

One study included carers of persons with either schizophrenia or bipolar disorder [56], and is therefore included in both Tables 1 and 2. One study included carers of persons with either bipolar disorder or substance use disorder [30], and is therefore included in both Tables 2 and 3.

A psychoeducational program was implemented in 11 studies, either as a single-family [70–72] or as a group approach [20, 75]. All studies except one [38] reported a positive effect of the intervention on considered outcomes (i.e., improvement of levels of burden, self-efficacy, and/or quality of life).

#### Efficacy of psychosocial interventions on primary and secondary outcomes in carers of persons with bipolar disorder

In the overall group of carers of persons with bipolar disorder receiving a psychosocial intervention, there was an improvement in the levels of personal burden (SMD: –1.15, 95% CI: –2.0 to –0.3, *p <* 0.005) and depressive symptoms (SMD: 3.70, 95% CI: 6.95 to 0.45, *p <* 0.005; Table 4). Risk of bias and indirectness were rated as not serious for all included outcomes. Inconsistency was rated as serious for all outcome measures. Imprecision was rated as serious for studies on well-being/quality of life, depressive symptoms, knowledge about the disorder, skills/coping skills, and self-efficacy (see Table 4). Certainty of evidence was rated as moderate for studies considering personal burden as outcome measure; low for studies considering depressive symptoms, knowledge about the disorder, skills/coping skills, and self-efficacy as outcome measures.

**Table 4.** GRADE table for psychosocial interventions compared to treatment as usual, usual psychiatric care, or waiting list for carers of persons with bipolar disorder

Certainty assessment							No. of patients		Effect		Certainty	Importance
No. of studies	Study design	Risk of bias	Inconsistency	Indirectness	Imprecision	Other considerations	Psychosocial interventions	Placebo	Relative (95% CI)	Absolute (95% CI)		
*Personal Burden (assessed with: Family Burden Self-Report Scale or Family Burden Interview Schedule or Family Problem Questionnaire or Burden Assessment Scale or Involvement Evaluation Questionnaire (IEQ))*												
7	Randomized trials	Not serious	Serious^a^	Not serious	Not serious	None	343	290	–	SMD **1.15** **SD lower** (2 lower to 0.3 lower)	⨁⨁⨁◯ Moderate	CRITICAL
*Wellbeing/quality of life (assessed with: Health Survey-36-Item Short Form or General Health Questionnaire-28 or Mental Health Continuum short form or Carer Wellbeing and Support Questionnaire or WHOQOL-BREF)*												
6	Randomized trials	Not serious	Serious^a^	Not serious	Serious^b^	None	518	439	–	SMD **1.08 SD higher** (0.27 lower to 2.44 higher)	⨁⨁◯◯ Low	CRITICAL
*Depressive symptoms (assessed with: Depression Anxiety Stress Scale (DASS))*												
3	Randomized trials	Not serious	Serious^a^	Not serious	Serious^c^	None	69	68	–	SMD **3.7 SD lower** (6.95 lower to 0.45 lower)	⨁⨁◯◯ Low	CRITICAL
*Knowledge about the disorder (assessed with: Mental Health Literacy Scale or ad-hoc scale on knowledge about the disorder or Brief Illness Perception Questionnaire or Knowledge of Illness Questionnaire (KOIQ) or Bipolar Disorder Knowledge Questionnaire)*												
4	Randomized trials	Not serious	Serious^a^	Not serious	Serious^b^ ^,^^c^	None	103	74	–	SMD **0.72 SD higher** (0.42 lower to 1.86 higher)	⨁⨁◯◯ Low	IMPORTANT
*Skills/coping skills (assessed with: Brief COPE or COPE or Family Problem Questionnaire)*												
3	Randomized trials	Not serious	Serious^a^	Not serious	Serious^b^	None	502	499	–	SMD **0.24 SD higher** (0.47 lower to 0.95 higher)	⨁⨁◯◯ Low	IMPORTANT
*Self-efficacy (assessed with: Ad-hoc Questionnaire or General Self-Efficacy Scale)*												
3	Randomized trials	Not serious	Serious^a^	Not serious	Serious^b^ ^,^^c^	None	61	73	–	SMD **1.42 SD higher** (0.29 lower to 3.14 higher)	⨁⨁◯◯ Low	IMPORTANT

CI: confidence interval; SMD: standardized mean difference.

a Severe, unexplained, heterogeneity (*I* ^2^ ≥ 60% or X^2^ < 0.05).

b Wide CI crossing the line of no effect.

c Less than 400 participants.

Bold characters indicates statistical significance at *p* < 0.05.

#### Subgroup analysis on efficacy of psychosocial interventions on carers of persons with bipolar disorder

A subgroup analysis based on the type of psychosocial intervention included in the different studies was performed (see Supplementary Table 2). Psychoeducational intervention was significantly associated with an improvement in the levels of personal burden (SMD: –0.63, 95% CI: –1.31 to –0.06, *p <* 0.005) and depressive symptoms (SMD: –3.70, 95% CI: –6.95 to –0.45, *p <* 0.005). Family-led supportive interventions were associated with an improvement in the levels of family burden (SMD: –4.03, 95% CI: 5.11 to 2.95, *p <* 0.005). Family-focused intervention and online “mi.spot” intervention were associated with a significant reduction in the levels of depressive symptoms (family-focused intervention, SMD: –5.46, 95% CI: –6.85 to 4.07, *p <* 0.005; “mi.spot,” SMD: –4.58, 95% CI: –10.40 to –1.24, *p <* 0.005). The remaining types of interventions were not associated with a statistically significant improvement in assessed outcomes.

### Studies on carers of persons with substance use disorders

Four studies on carers of persons with substance use disorders were identified. All details are reported in Table 5. The studies were RCTs [27, 29] or pre-test/post-test trials [27, 30]. All studies adopted validated assessment tools. One study included carers of persons with both bipolar disorder or substance use disorder [30], and is therefore included in both Tables 3 and 5. Two studies [27, 29] adopted an educational/informative approach.

**Table 5.** Studies on carers of persons with substance use disorder (*n =* 4)

Author(s), country (year)	Sample and design	Intervention	Main components of intervention(s)	Assessment instruments	Main results in the experimental group	Global comment
Karimi et al., Iran (2019) [29]	*N =* 80 spouses (*N =* 40 experimental intervention; *N =* 40 control group) Randomized controlled trial	Supportive-educational intervention versus control group not receiving any training	Quality of life therapy	Depression Anxiety and Stress Scale (DASS-21) Satisfaction with Life Scale (SWLS)	Improvement in the levels of life satisfaction.	Positive effect
Hojjat et al., Iran (2016) [27]	*N =* 48 wives (*N =* 23 experimental intervention; *N =* 25 control group) Pre-test/post-test	Educational group program versus waiting list	Information about disorder and its treatment Harm reduction Relapse prevention	ENRICH (Evaluation and Nurturing Relationship Issues, Communication, and Happiness) Marital Satisfaction (EMS) Scale – short form	Improvement in the levels of marital satisfaction.	Positive effect
Osilla et al., USA (2017) [28]	*N =* 312 spouses (*N =* 162 experimental intervention; *N =* 150 control condition) Randomized controlled trial	Partners Connect (web-based intervention using behavioral skills such as self-care and healthy communication) versus waiting list	Motivational interviewing Cognitive behavioral therapy strategies Self-care skills	Social Support Survey (SSS) Family Environment Scale (FES)	Reduction in levels of anxiety and improvement in levels of emotional/informational and social support at follow-up.	Positive effect
Reupert et al., Australia (2018) [30]	*N =* 31 children (aged 18–25 years) with a parent having mental illness and/or substance use disorder Pre-test/post-test	Online intervention (“mi.spot”) targeting cognitive reappraisal, connectedness to others, and resilience	Information about the disorder Coping strategies	Mental Health Continuum short form (MHC-SF) Depression Anxiety and Stress Scale (DASS-21) Coping Orientation to Problems Experienced (COPE) inventory General Help-Seeking Questionnaire (GHSQ) Social Connectedness Scale (SCS) Mental Health Literacy Scale (MHLS) General Self-Efficacy Scale (GSE)	Improvement in depressive symptoms, stress levels, well-being and autonomy.	Positive effect

#### Efficacy of psychosocial interventions on primary and secondary outcomes in carers of persons with bipolar disorder

In the overall group of carers of persons with substance use disorder receiving any psychosocial intervention, there was an improvement in the levels of well-being (SMD: 0.85, 95% CI: 0.40 to 1.31, *p <* 0.005; Table 6). Risk of bias was not serious for studies including well-being/quality of life and depressive symptoms as outcome measures, whereas it was rated as very serious for studies considering knowledge about the disorder, skills/coping skills, and self-efficacy. Inconsistency and indirectness were rated as serious for all outcome measures. Imprecision was rated as serious for all outcome measures (see Table 6). Certainty of evidence was rated as low for studies considering well-being/quality of life and depressive symptoms as outcome measures; and as very low for studies considering knowledge about the disorder, skills/coping skills, and self-efficacy as outcome measures. No data were available on levels of personal burden.

**Table 6.** GRADE table for psychosocial interventions compared to treatment as usual, usual psychiatric care, or waiting list for carers of persons with substance use disorders

Certainty assessment							No. of patients		Effect		Certainty	Importance
No. of studies	Study design	Risk of bias	Inconsistency	Indirectness	Imprecision	Other considerations	Psychosocial interventions	Placebo	Relative (95% CI)	Absolute (95% CI)		
*Personal Burden*												
None									not estimable		–	CRITICAL
*Wellbeing/quality of life (assessed with: Mental Health Continuum short form or Carer Wellbeing and Satisfaction with Life Scale)*												
1	Randomized trial	Not serious	Serious^a^	Not serious	Serious^b^ ^,^^c^	None	71	40	–	SMD **0.85** **SD higher** (0.4 higher to 1.31 higher)	⨁⨁◯◯ Low	CRITICAL
*Depressive symptoms (assessed with: Depression Anxiety Stress Scale (DASS))*												
3	Randomized trials	Not serious	Serious^a^	Not serious	Serious^b^ ^,^^c^	None	169	207	–	SMD **0.25 SD lower** (0.85 lower to 0.35 higher)	⨁⨁◯◯ Low	CRITICAL
*Knowledge about the disorder (assessed with: Mental Health Literacy Scale)*												
1	Observational studies	Very serious	Serious^a^	Not serious	Serious^b^ ^,^^c^	None	31	0	–	MD **0.09 higher** (8.73 lower to 8.91 higher)	⨁◯◯◯ Very low	IMPORTANT
*Skills/coping skills (assessed with: COPE)*												
1	Observational studies	Very serious	Serious^a^	Not serious	Serious^b^ ^,^^c^	None	31	0	–	MD **0.04 SD higher** (0.46 lower to 0.54 higher)	⨁◯◯◯ Very low	IMPORTANT
*Self-efficacy (assessed with: General Self-Efficacy Scale)*												
1	Observational studies	Very serious	Serious^a^	Not serious	Serious^b^ ^,^^c^	None	31	0	–	MD **2.38 SD higher** (5.52 lower to 10.8 higher)	⨁◯◯◯ Very low^f^	IMPORTANT

CI: confidence interval; MD: mean difference; SMD: standardized mean difference.

a Severe, unexplained, heterogeneity (*I* ^2^ ≥ 60% or X^2^ < 0.05).

b Wide CI crossing the line of no effect.

c Less than 400 participants.

Bold characters indicates statistical significance at *p* < 0.05.

#### Subgroup analysis on efficacy of psychosocial interventions on carers of persons with substance use disorders

A subgroup analysis based on the type of psychosocial interventions included in the different studies was performed (see Supplementary Table 3). Due to the low number of trials available for carers of persons with substance use disorders, this analysis did not detect any statistically significant difference between the various psychosocial interventions.

## Discussion

The present systematic review and meta-analysis suggests that several psychosocial interventions are effective in carers of persons with schizophrenia/psychosis/schizophrenia spectrum disorder and bipolar disorder, with a moderate to low level of certainty. In particular, in carers of persons with schizophrenia/psychosis/schizophrenia spectrum disorder, psychoeducational interventions are significantly associated with an improvement in personal burden, well-being, and knowledge about the illness; and a supportive-educational intervention with an improvement in personal burden. In carers of persons with bipolar disorder, a psychoeducational intervention is significantly associated with an improvement in personal burden and depressive symptoms; family-led supportive interventions with an improvement in family burden; family-focused intervention and online “mi-spot” intervention with a significant reduction in depressive symptoms. Available studies focusing on carers of persons with substance use disorders found that psychosocial interventions used in this population are overall effective only on the level of well-being.

The psychoeducational approach is the most frequent intervention provided to carers of people with severe mental disorders, although different psychoeducational approaches exist. Common elements to the different approaches include the provision of problem-solving techniques [34, 39, 40, 44–46, 52, 58, 59, 70–75, 81, 82, 100]; the promotion of appropriate coping strategies [20, 24, 30, 34, 42, 51, 58, 66, 67, 73, 82, 95, 96]; and the teaching of communication skills [51, 52, 57, 58, 63, 67, 69, 82, 97–99]. This intervention can be delivered in different settings (i.e., at the mental health center, at home), and format (individual vs. group; Table 7). The heterogeneity of the different psychoeducational approaches may represent a bias in the evaluation of their efficacy. In fact, differences include duration of the intervention, involvement of different mental health professionals, inclusion of the patients, etc. It has to be noted that short-term interventions, with an active involvement of users and carers in the provision of the intervention and the inclusion of booster sessions have been associated with higher levels of improvements of the considered outcomes. Another limitation of our review is that the majority of studies have been carried out in high-income countries, while only a few data are available from low- and middle-income countries (LMICs).

**Table 7.** Core features of psychoeducational approaches

Commonalities	Differences
Teaching of problem-solving techniques	Type of settings: at the mental health center, at home
Promotion of appropriate coping strategies	Format: individual, group, single family, multiple families
Teaching of communication skills	Number of sessions

The dissemination of psychoeducation in ordinary routine practice is hampered by several obstacles, such as lack of training for health care professionals, lack of time for running the intervention, and lack of interest by carers [94]. Thus, research should focus on innovative strategies to promote the dissemination of psychoeducational interventions for carers of people with severe mental disorders on a larger scale, including web-based or app-based approach, such as the one included in the “mi.spot” intervention [83]. However, few studies have been conducted so far in LMICs, highlighting the need to further promote the dissemination of such psychosocial interventions in those contexts [84, 85]. It should be that those psychosocial interventions need to be adapted to the socio-cultural context and to the limited resources of such countries, but their efficacy should not be impacted. However, further studies are needed and the mhGAP can be very important for supporting the dissemination of these interventions in LMIC settings [77, 86].

The evidence concerning the interventions for carers of people with substance use disorders is very limited, with only four detected studies. Carers of people with these disorders report specific needs in terms of emotional and practical support, which would deserve targeted investigation. Furthermore, the increasing prevalence of these disorders, in particular in the young population [87], further highlights the need to develop supportive interventions for carers [78, 88].

Another encouraging finding is that online “mi-spot” intervention is effective in reducing levels of depressive symptoms in carers of patients with bipolar disorder, confirming the potential applicability and clinical utility of web-based and app-based interventions in the mental health field. In recent years, telepsychiatry and tele-mental health has witnessed an exponential growth, further nurtured by the COVID-19 pandemic and by the need to reduce in-person contact [89, 90]. A recent systematic review and meta-analysis on technology-based supportive intervention for carers of people with dementia has found that such approaches are beneficial for carers, especially in terms of increased accessibility overtime [91]. However, digital interventions are still in their infancy regarding applicability as supportive interventions for carers of people with severe mental disorders, although these preliminary findings are encouraging [79, 80, 92].

Furthermore, the majority of included studies (*n =* 48 studies; 72.7%) adopted a randomized controlled design, which represents the most rigorous methodological approach for experimental trials. However, some studies, especially those conducted in LMICs have been carried out using less rigorous methodologies, including pre-/post-evaluations (without control groups), small sample sizes with high attrition rates, using different outcome measures, and with different duration of the interventions (ranging from single session intervention to 6-month interventions). Of course, these methodological differences have been carefully taken into account when evaluating the global level of certainty of data. While the evidence from high-income countries is rather robust, there is the need to promote well-structured and rigorous studies in LMIC, to increase the quality of evidence.

Our study has some limitations. First, the quality of the evidence ranges from very low to moderate. For example, in studies on carers of people with schizophrenia, the risk of bias was rated as serious for several outcomes, such as the levels of knowledge or self-efficacy, being based on just one observational study. Moreover, the included studies are characterized by extreme heterogeneity, lack of precision, and small sample sizes, which might significantly affect statistical analyses. Another limitation is related to the type of considered outcomes, which are measured only using self-reported assessment tools, with a potential risk of response bias.

Carers of patients suffering from schizophrenia, bipolar disorder, and substance use disorders report different clinical unmet needs and different types of objective and subjective burden, reflecting the clinical heterogeneity of such disorders. The decision to include these three diagnostic categories has been due to the need to update the mhGAP focusing on the most burdensome disorders for patients and their carers. Moreover, we have tried to overcome this possible bias by carrying out subgroup analysis on the different models of psychosocial interventions.

## Conclusions

Our analysis confirms that several psychosocial interventions are effective in supporting carers of people with severe mental disorders. However, there is a need to collect more data of good quality, particularly in LMIC. Moreover, the efficacy and sustainability of those interventions should be evaluated in longer-term studies carried out in the real world.

## Supporting information

Sampogna et al. supplementary material Sampogna et al. supplementary material

## Supplementary material

The supplementary material for this article can be found at http://doi.org/10.1192/j.eurpsy.2023.2472.

## Appendix 1 – Description of psychosocial interventions

### Brief Cognitive Behavioral Stress Management Program

The Brief Cognitive Behavioral Stress Management Program is a semi-structured program that was devised to allow the caregiver to gain awareness of various aspects of his or her daily life and distress due to caregiving responsibilities, as well as to assist the caregiver in developing stress management skills in a practical group environment. In this program, several aspects of other stress management programs, cognitive-behavioral therapy techniques, and resources for group psychotherapy are used. The approach has been manualized and consists of seven sessions.

### Collective Narrative Therapy

Collective Narrative Therapy is a narrative group intervention, consisting of eight group sessions with different goals. All groups are facilitated by the same practitioner. The main topics are: creating relationship; describe, externalize and evaluate problems; understand and solve problems; reflect and make sense of experiences, and share positive communication; relax body and mind, and explore the meaning of life and inner strengths; and rewrite life stories and ascertain life goals.

### Psychoeducation

Psychoeducation is defined as a psychosocial intervention with systematic and structured knowledge transfer about an illness and its treatment, integrating emotional and motivational aspects to enable carers to cope with the illness and to improve patients’ treatment adherence. The content of psychoeducation interventions includes etiology of illness, treatment process, adverse effects of prescribed medications, coping strategies, coping skills training, problem-solving training.

### Family-Focused Culturally-Informed Treatment model

The Family-Focused Culturally-Informed Treatment model is an individual or couple treatment for caregivers of persons with bipolar disorder. It is based on three premises supported by extensive research and theory: a) negative and dysfunctional automatic thoughts, feelings, and core beliefs about caregiving contribute to and sustain depressive symptoms and perceived burden among caregivers; b) depressive symptoms interfere with caregiver self-care and ability to manage the demands and stress associated with caregiving; c) the presence of caregiver’s depressive symptoms interferes with management of caregiving demands and impacts on severity of patient’s mood symptoms.

### Family-Led Mutual Support Group program

The Family-Led Mutual Support Group program consists of 16 bi-weekly 2-h sessions co-led by two peer family caregivers. These caregivers are relatively more experienced in caregiving and are trained by the researchers to perform the peer leader role with a three full-day psychoeducation and supportive skills workshop. The workshop’s contents are structured in five stages, including engagement; awareness and addressing mutually shared psychosocial needs; managing common and individual physical and psychosocial needs of self and family members; taking up caregiving roles and demands and facing challenges; group termination.

All sessions place emphasis on supportive sharing of experience and information exchanges, problem-solving, and caregiving skill practices.

### Yoga/Self-Help intervention

This consists of a self-help manual and DVD for practicing yoga intended for caregivers of patients with schizophrenia.

### Supportive-Educational interventions

Supportive-Educational interventions consist of sessions aiming to improve levels of knowledge about the disorder, how to handle difficult behaviors, stress management, communication skills, and relapse prevention.

### Online intervention (“mi.spot”)

“mi.spot” is an online, manualized intervention that targets young adults who have a parent with a mental illness and/or substance use disorder. The topics include: introduction to the intervention; information about mental disorders; assessing relationship with parents and/or other family members; managing stress; discussion on caring responsibilities; taking control of own life.
