# Supplementary material for: Psychosocial interventions for carers of people with severe mental and substance use disorders: a systematic review and meta-analysis
Source: Eur Psychiatry. 2023 Nov 24;66(1):e98. doi: 10.1192/j.eurpsy.2023.2472 (PMC10755580; doi:10.1192/j.eurpsy.2023.2472)
Supplement: Sampogna et al. supplementary material [file S0924933823024720sup001.docx]

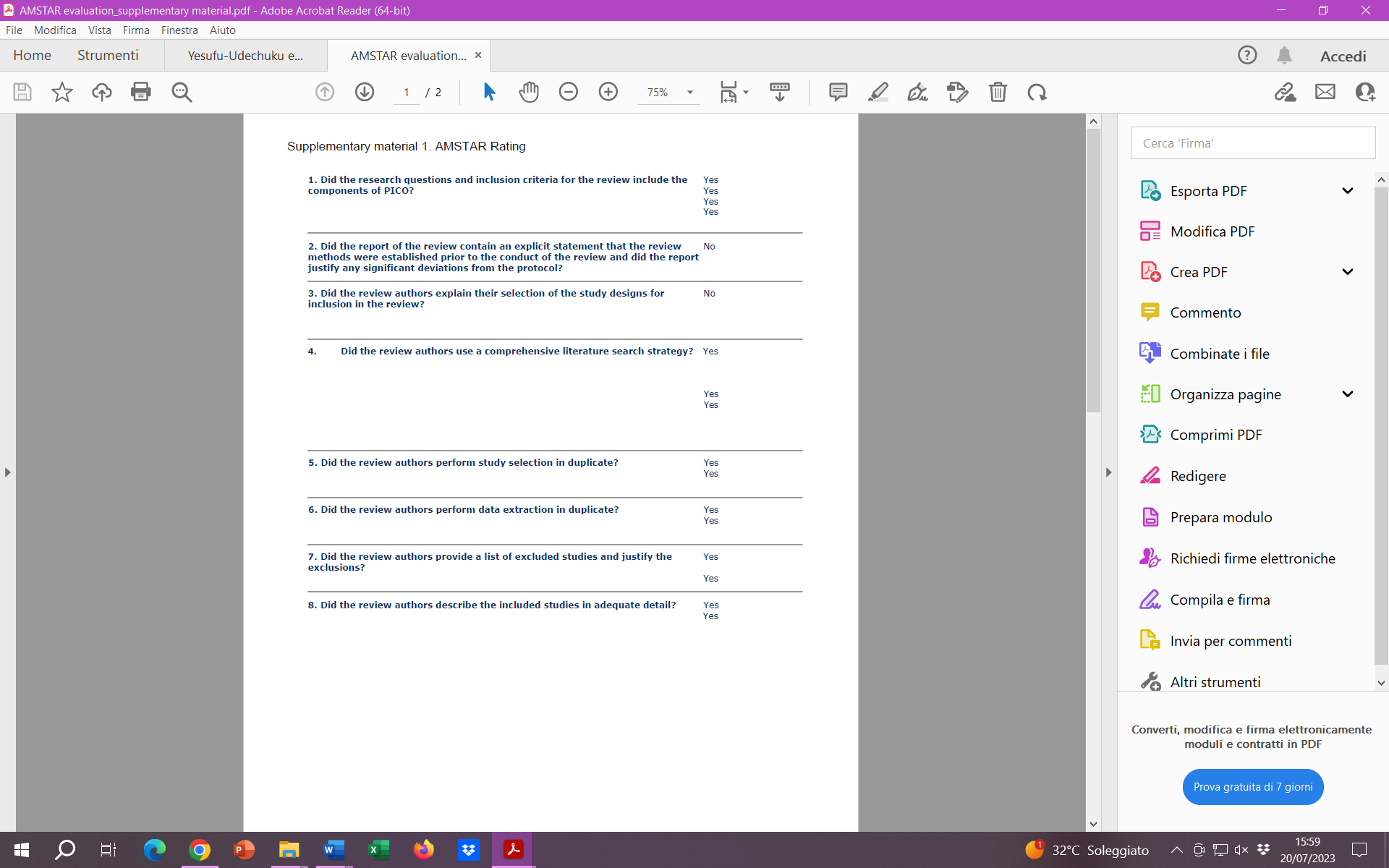

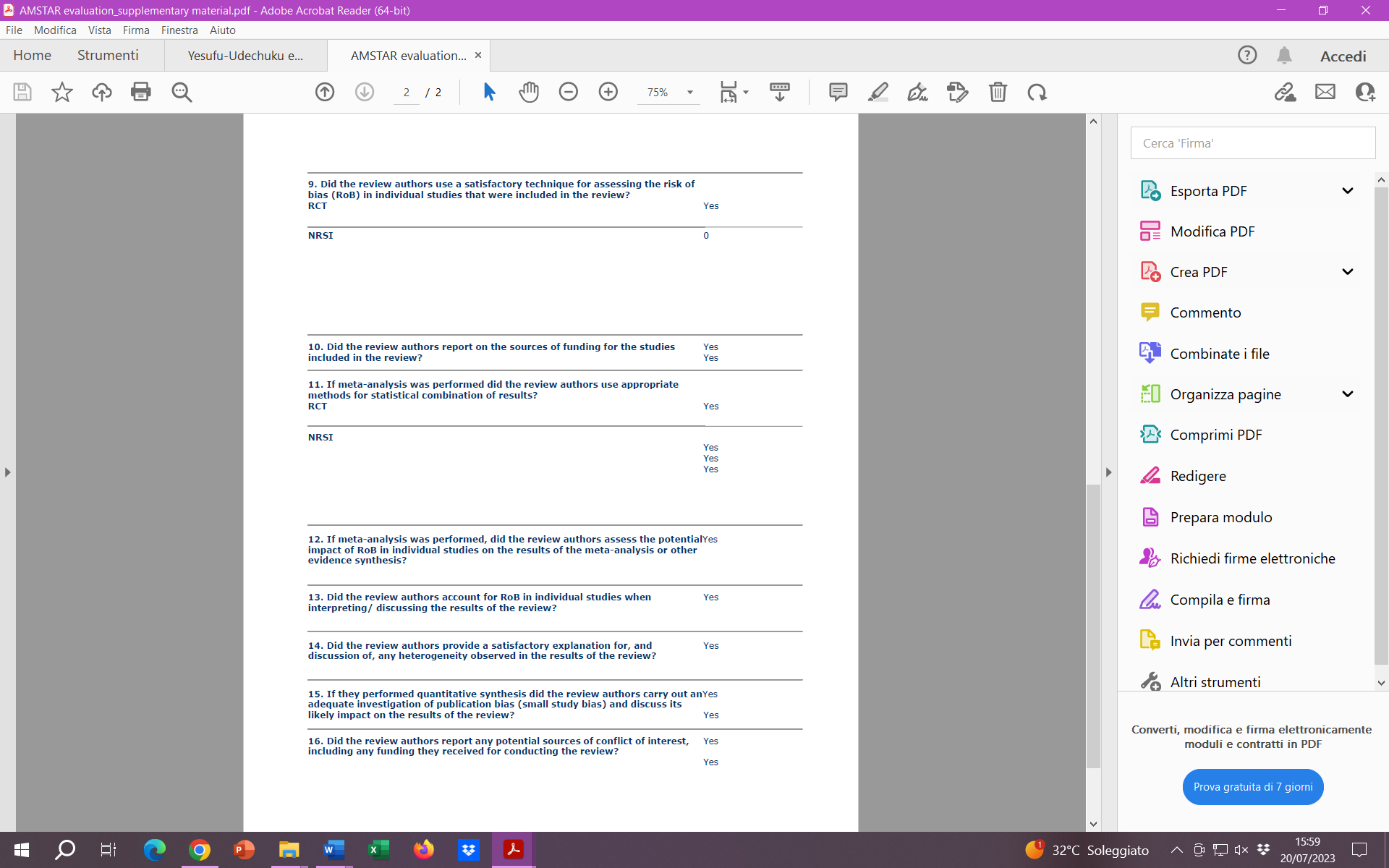


| Supplementary Table 1 | | | | | |
| --- | --- | --- | --- | --- | --- |
| GRADE Table | Outcome | Subgroup analysis | Number of Studies | Effects | Certainty of Evidence |
| **Grade Table 1:**  Psychosocial interventions compared to treatment as usual, usual psychiatric care, or waiting list for carers of persons with psychosis | Personal burden | - | 22 | **SMD 0.61 lower*** (0.86 lower to 0.36 lower) | ⨁⨁⨁◯  Moderate |
|  |  | Psychoeducation | 13 | SMD 0.70 lower*  (1.01 lower to 0.40 lower) | ⨁⨁⨁◯  Moderate |
|  |  | Supportive-Educational interventions | 2 | SMD 0.26 lower*  (0.67 lower to 0.14 higher) | ⨁⨁◯◯ Low |
|  |  | Stress management | 1 | MD 0.73 lower*  (1.25 lower to 0.21 lower) | ⨁◯◯◯  Very low |
|  |  | Collective Narrative Therapy | 1 | - |  |
|  |  | Family-Led Mutual Support | 3 | SMD 0.72 lower  (1.73 lower to 0.29 higher) | ⨁⨁◯◯ Low |
|  |  | Yoga intervention | 1 | MD 0.29  (0.28 lower to 0.36 lower) | ⨁◯◯◯  Very low |
|  | Well-being/Quality of life | - | 18 | **SMD 0.72 higher*** (0.39 higher to 1.05 higher) | ⨁⨁⨁◯  Moderate |
|  |  | Psychoeducation | 10 | SMD 1.04 higher  (0.53 higher to 1.54 higher) | ⨁⨁⨁◯  Moderate |
|  |  | Supportive-Educational interventions | 2 | SMD 0.13 higher  (0.70 lower to 0.97 higher) | ⨁⨁◯◯ Low |
|  |  | Stress management | 1 | MD 0.41 higher  (0.10 lower to 0.92 higher) | ⨁◯◯◯  Very low |
|  |  | Collective Narrative Therapy | 1 | MD 0.38 higher  (0.12 lower to 0.87 higher) | ⨁◯◯◯  Very low |
|  |  | Family-Led Mutual Support | 3 | SMD 0.88 higher  (0.46 higher to 1.29 higher) | ⨁⨁◯◯ Low |
|  |  | Yoga intervention | 1 | MD 0.27 higher  (0.30 lower to 0.84 higher) | ⨁◯◯◯  Very low |
|  | Depressive symptoms | - | 6 | **SMD 0.76 lower** (1.61 lower to 0.1 higher) | ⨁⨁◯◯ Low |
|  |  | Psychoeducation | 1 | MD 1.57 lower  (1.98 lower to 1.17 lower) | ⨁◯◯◯  Very low |
|  |  | Supportive-Educational interventions | 1 | MD 0.33 lower  (0.66 lower to 0.00 lower) | ⨁◯◯◯  Very low |
|  |  | Stress management |  | - |  |
|  |  | Collective Narrative Therapy |  | - |  |
|  |  | Family-Led Mutual Support |  | - |  |
|  |  | Yoga intervention |  | - |  |
|  | Knowledge about the disorder | - | 7 | **SMD 0.6 higher*** (0.2 higher to 1.01 higher) | ⨁⨁◯◯ Low |
|  |  | Psychoeducation | 4 | SMD 0.65 higher*****  (0.30 higher to 0.99 higher) | ⨁⨁◯◯ Low |
|  |  | Supportive-Educational interventions | 3 | SMD 0.61  (0.40 lower to 1.62 higher) | ⨁⨁◯◯ Low |
|  |  | Stress management |  | **-** |  |
|  |  | Collective Narrative Therapy |  | **-** |  |
|  |  | Family-Led Mutual Support |  | **-** |  |
|  |  | Yoga intervention |  | **-** |  |
|  | Skills/coping skills | - | 8 | **SMD 0.10 higher** (0.21 lower to 0.41 higher) | ⨁⨁◯◯  Low |
|  |  | Psychoeducation | 6 | SMD 0.17 higher  (0.19 lower to 0.52 higher) | ⨁⨁◯◯  Low |
|  |  | Supportive-Educational interventions | 2 | SMD 0.45 lower  (0.94 lower to 0.05 higher) | ⨁⨁◯◯  Low |
|  |  | Stress management | 1 | MD 0.73 higher*  (0.21 higher to 1.25 higher) | ⨁◯◯◯  Very low |
|  |  | Collective Narrative Therapy |  | **-** |  |
|  |  | Family-Led Mutual Support |  | **-** |  |
|  |  | Yoga intervention |  | **-** |  |
|  | Self-efficacy | - | 2 | **SMD 1.15 higher** (6.16 lower to 8.46 higher) | ⨁◯◯◯ Very Low |
|  |  | Psychoeducation | 2 | SMD 1.15 higher (6.16 lower to 8.46 higher) | ⨁◯◯◯  Very Low |
|  |  | Supportive-Educational interventions |  | **-** |  |
|  |  | Stress management |  | **-** |  |
|  |  | Collective Narrative Therapy |  | **-** |  |
|  |  | Family-Led Mutual Support |  | **-** |  |
|  |  | Yoga intervention |  | **-** |  |

*Statistically significant

| Supplementary Table 2 | | | | | |
| --- | --- | --- | --- | --- | --- |
| GRADE Table | Outcome | Subgroup analysis | Number of Studies | Effects | Certainty of Evidence |
| **Grade Table 2:**  Psychosocial interventions compared to treatment as usual, usual psychiatric care, or waiting list for carers of persons with bipolar disorder | Personal burden | - | 7 | **SMD 1.15 lower*** (2 lower to 0.3 lower) | ⨁⨁⨁◯  Moderate |
|  | Psychoeducation | Psychoeducation | 6 | SMD 0.63 lower*  (1.31 lower to 0.06 lower) | ⨁⨁⨁◯  Moderate |
|  |  | Supportive-Educational interventions | - | - |  |
|  |  | Stress management | -- | - |  |
|  |  | Collective Narrative Therapy | - | - |  |
|  |  | Family-Led Mutual Support | 1 | MD 4.03 lower*  (5.11 lower to 2.95 lower) | ⨁◯◯◯  Very low |
|  |  | Yoga intervention | - | - |  |
|  |  | Family focused intervention | - | - |  |
|  |  | Online intervention (“mi.spot”) | - | - |  |
|  | Well-being/quality of life | - | 6 | **SMD 1.08 higher** (0.27 lower to 2.44 higher) | ⨁⨁◯◯  Low |
|  |  | Psychoeducation | 5 | SMD 0.27 higher  (0.22 lower to 0.76 higher) | ⨁⨁◯◯  Low |
|  |  | Supportive-Educational interventions |  | - |  |
|  |  | Stress management |  | - |  |
|  |  | Collective Narrative Therapy |  | - |  |
|  |  | Family-Led Mutual Support |  | - |  |
|  |  | Yoga intervention |  | - |  |
|  |  | Family focused intervention | 1 | MD 2.62 higher*  (1.78 higher to 3.46 higher) | ⨁◯◯◯  Very low |
|  |  | Online intervention (“mi.spot”) |  | - |  |
|  | Depressive symptoms | - | 3 | **SMD 3.70 lower*** (6.95 lower to 0.45 lower) | ⨁⨁◯◯ Low |
|  |  | Psychoeducation | 1 | MD 1.47 lower*  (3.18 lower to 0.24 lower) | ⨁◯◯◯  Very Low |
|  |  | Supportive-Educational interventions |  | - |  |
|  |  | Stress management |  | - |  |
|  |  | Collective Narrative Therapy |  | - |  |
|  |  | Family-Led Mutual Support |  | - |  |
|  |  | Yoga intervention |  | - |  |
|  |  | Family focused intervention | 1 | MD 5.46 lower*  (6.85 lower to 4.07 lower) | ⨁◯◯◯  Very Low |
|  |  | Online intervention (“mi.spot”) | 1 | MD 4.58 lower*  (10.40 lower to 1.24 lower) | ⨁◯◯◯  Very Low |
|  | Knowledge about the disorder | - | 4 | **SMD 0.72 higher** (0.42 lower to 1.86 higher) | ⨁⨁◯◯ Low |
|  |  | Psychoeducation | 2 | SMD 0.98 higher  (0.63 lower to 2.58 higher) | ⨁⨁◯◯ Low |
|  |  | Supportive-Educational interventions |  | - |  |
|  |  | Stress management |  | **-** |  |
|  |  | Collective Narrative Therapy |  | **-** |  |
|  |  | Family-Led Mutual Support |  | **-** |  |
|  |  | Yoga intervention |  | **-** |  |
|  |  | Family focused intervention |  | **-** |  |
|  |  | Online intervention (“mi.spot”) | 1 | MD 0.01 higher  (0.49 lower to 0.50 higher) | ⨁◯◯◯  Very Low |
|  | Skills/coping skills | - | 3 | **SMD 0.24 higher** (0.47 lower to 0.95 higher) | ⨁⨁◯◯ Low |
|  |  | Psychoeducation | 2 | SMD 0.34 higher  (0.71 lower to 1.38 higher) | ⨁⨁◯◯ Low |
|  |  | Supportive-Educational interventions |  | - |  |
|  |  | Stress management |  | - |  |
|  |  | Collective Narrative Therapy |  | **-** |  |
|  |  | Family-Led Mutual Support |  | **-** |  |
|  |  | Yoga intervention |  | **-** |  |
|  |  | Family focused intervention |  | **-** |  |
|  |  | Online intervention (“mi.spot”) | 1 | MD 0.04 higher  (0.46 lower to 0.54 higher) | ⨁◯◯◯  Very Low |
|  | Self-efficacy | - | 3 | **SMD 1.42 higher** (0.29 lower to 3.14 higher) | ⨁⨁◯◯  Low |
|  |  | Psychoeducation | 2 | SMD 2.22 higher (1.62 lower to 6.05 higher) | ⨁⨁◯◯  Low |
|  |  | Supportive-Educational interventions |  | **-** |  |
|  |  | Stress management |  | **-** |  |
|  |  | Collective Narrative Therapy |  | **-** |  |
|  |  | Family-Led Mutual Support |  | **-** |  |
|  |  | Yoga intervention |  | **-** |  |
|  |  | Family focused intervention |  | **-** |  |
|  |  | Online intervention (“mi.spot”) | 1 | MD 0.25 higher  (0.35 lower to 0.65 higher) | ⨁◯◯◯  Very Low |

| Supplementary Table 3 | | | | | |
| --- | --- | --- | --- | --- | --- |
| GRADE Table | Outcome | Subgroup analysis | Number of Studies | Effects | Certainty of Evidence |
| **Grade Table 3:**    Psychosocial interventions compared to treatment as usual, usual psychiatric care, or waiting list for carers of persons with substance use disorders | Personal burden | - |  | - |  |
|  |  | Psychoeducation |  | - |  |
|  |  | Supportive-Educational interventions |  | - |  |
|  |  | Stress management |  | - |  |
|  |  | Mutual support |  | - |  |
|  |  | Yoga intervention |  | - |  |
|  |  | Family focused intervention |  | - |  |
|  |  | Online intervention |  | - |  |
|  | Well-being/quality of life | - | 2 | **SMD 0.85 higher*** (0.4 higher to 1.31 higher) | ⨁⨁◯◯ Low |
|  |  | Psychoeducation |  | - |  |
|  |  | Supportive-Educational interventions | 1 | MD 0.85 higher  (0.40 higher to 1.31 higher) | ⨁◯◯◯ Very Low |
|  |  | Stress management |  | - |  |
|  |  | Collective Narrative Therapy |  | - |  |
|  |  | Family-Led Mutual Support |  | - |  |
|  |  | Yoga intervention |  | - |  |
|  |  | Family focused intervention |  | - |  |
|  |  | Online intervention | 1 | MD 0.01 higher  (0.50 lower to 0.50 higher) |  |
|  | Depressive symptoms | - | 3 | **SMD 0.25 lower** (0.85 lower to 0.35 higher) | ⨁◯◯◯ Very Low |
|  |  | Psychoeducation |  | - |  |
|  |  | Supportive-Educational interventions | 2 | MD 0.67 lower  (1.13 lower to 0.22 lower) | ⨁◯◯◯  Very low |
|  |  | Stress management |  | - |  |
|  |  | Collective Narrative Therapy |  | - |  |
|  |  | Yoga intervention |  | - |  |
|  |  | Family-Led Mutual Support |  | - |  |
|  |  | Online intervention (“mi.spot”) | 1 | MD 0.04 lower  (0.64 lower to 0.56 lower) | ⨁◯◯◯  Very low |
|  | Knowledge about the disorder | - | 1 | **MD 0.09 higher** (8.73 lower to 8.91 higher) | ⨁◯◯◯  Very low |
|  |  | Psychoeducation |  | - |  |
|  |  | Supportive-Educational interventions |  | - |  |
|  |  | Stress management |  | **-** |  |
|  |  | Collective Narrative Therapy |  | **-** |  |
|  |  | Yoga intervention |  | **-** |  |
|  |  | Family-Led Mutual Support |  | **-** |  |
|  |  | Online intervention (“mi.spot”) | 1 | MD 0.09 higher (8.73 lower to 8.91 higher) | ⨁◯◯◯  Very low |
|  | Skills/coping skills | - | 1 | **MD 0.04 higher** (0.46 lower to 0.54 higher) | ⨁◯◯◯  Very low |
|  |  | Psychoeducation |  |  |  |
|  |  | Informative/educational information |  | - |  |
|  |  | Stress management |  | - |  |
|  |  | Collective Narrative Therapy |  | **-** |  |
|  |  | Mutual support |  | **-** |  |
|  |  | Yoga intervention |  | **-** |  |
|  |  | Family focused intervention |  | **-** |  |
|  |  | Online intervention (“mi.spot”) | 1 | MD 0.04 higher (0.46 lower to 0.54 higher) | ⨁◯◯◯  Very low |
|  | Self-efficacy | - | 1 | **MD 2.38 higher** (5.52 lower to 10.8 higher) | ⨁◯◯◯  Very low |
|  |  | Psychoeducation |  | - |  |
|  |  | Informative/educational information |  | **-** |  |
|  |  | Stress management |  | **-** |  |
|  |  | Collective Narrative Therapy |  | **-** |  |
|  |  | Mutual support |  | **-** |  |
|  |  | Yoga intervention |  | **-** |  |
|  |  | Family-Led Mutual Support |  | **-** |  |
|  |  | Online intervention (“mi.spot”) | 1 | MD 2.38 higher (5.52 lower to 10.8 higher) | ⨁◯◯◯  Very low |
